# Activation of Inflammatory Responses Correlate With Hedgehog Activation and Precede Expansion of Cancer Stem-Like Cells in an Animal Model of Residual Triple Negative Breast Cancer after Neoadjuvant Chemotherapy

**DOI:** 10.17140/CSMMOJ-2-112

**Published:** 2015-12-07

**Authors:** Kimberly M. Arnold, Nicole J. Flynn, Jennifer Sims-Mourtada

**Affiliations:** 1 Center for Translational Cancer Research, Helen F. Graham Cancer Center, Christiana Care Health Services, Inc., Newark, DE, USA; 2 Department of Medical Laboratory Sciences, University of Delaware, Newark, DE, USA; 3 Department of Biological Sciences, University of Delaware, Newark, DE, USA

**Keywords:** Hedgehog signaling, Inflammatory cytokines, Neoadjuvant therapy, Triple negative breast cancer

## Abstract

Triple Negative Breast Cancer (TNBC) is characterized as a lack of expression of the hormonal receptors, estrogen and progesterone, and Human epidermal growth factor receptor 2 (HER2) and as such is unresponsive to current targeted therapy. Resistance of breast cancers to treatment is thought to be due to a sub-population of tumor cells called Breast Cancer Stem Cells (BCSCs) and contributes to poor prognosis and increased risk of recurrence. Previously, we have shown that hedgehog activation is induced by chemotherapy and promotes expansion of a stem-like population in breast cancer cell lines. In addition, chemotherapy can induce an inflammatory response and inflammatory factors can lead to activation of Hedgehog (HH) at sites of tissue injury. Therefore, we wanted to investigate how chemotherapy altered hedgehog signaling and correlated with the release of inflammatory cytokines in a mouse model of breast cancer. Patient derived triple negative breast tumor bearing mice were treated with weekly doses of docetaxel. Following treatment, tumor volume decreased reaching a nadir around 15 days after the start of treatment and increased back to pre-treatment size 35-39 days post treatment. Immunohistochemical staining of mice tumors revealed that Sonic hedgehog and nuclear Gli-1 expression transiently increased following docetaxel treatment, reached peak expression at day 8, and subsequently decreased to almost pre-treatment levels following regrowth of the tumor. Similarly, Interleukin 6 (IL-6) and Interleukin 8 (IL-8) expression transiently increased, peaked around day 8, and decreased upon tumor regrowth, however, remained above pre-treatment levels. Expression of the stem cell marker ALDH1A3 proceeded activation of hedgehog signaling and expression of inflammatory cytokines, increasing around day 15 post treatment and continued to be elevated during tumor regrowth. Thus, chemotherapy treatment resulted in activation of the hedgehog pathway and release of inflammatory cytokines leading to long-term expansion of ALDH1A3 positive stem cells, which can contribute to the regrowth of the tumor and promote resistance to treatment.

## INTRODUCTION

Triple Negative Breast Cancer (TNBC) is an aggressive cancer defined by the lack of expression of Human epidermal growth factor receptor 2 (HER2) and the hormone receptors, estrogen and progesterone. Due to the receptor status, there are currently no targeted treatments that exist for this subset of breast cancers. Standard treatment for locally advanced TNBCs consists of Neoadjuvant chemotherapy (NCT) prior to surgical resection, followed by radiation and additional chemotherapy cycles. However, despite current interventions, TNBC are associated with poor prognosis and early visceral metastasis.^1^ Recently it has been suggested that pathological complete response to NCT can be used as a surrogate endpoint for prediction of long-term clinical benefit of systemic chemotherapy treatment. Patients who have pathological complete response have better overall survival and relapse free survival than those who do not.^2^ Currently there are no reliable biomarkers to predict which patients will respond to NCT and the mechanisms of resistance to this treatment are not fully understood.

Resistant breast cancers are reported to have an increase in a population of cells, known as Breast Cancer Stem Cells (BCSC), that resemble mammary stem cells.^3^ These cells are highly tumorigenic, have increased expression of survival pathways, Deoxyribonucleic acid (DNA) repair enzymes and resistance pathways. Additionally, they have increased activation of epithelial-mesenchymal transition pathways and have a greater metastatic potential. BCSC are characterized by high levels of Aldehyde dehydrogenase (ALDH) activity and increased expression of ALDH isoforms.^4^ In particular, expression of the ALDH isoform ALDH1A3 is inversely correlated with estrogen receptor signaling^5,6^ and may be predictive of metastatic potential in invasive breast cancers.^7^

The re-activation of developmental pathways has been implicated to play a role in the development and progression of cancer. In particular, abnormal regulation of the Hedgehog (HH) pathway can alter cellular proliferation and differentiation leading to tumorigenesis. Canonical HH signaling is induced by binding of the ligand Sonic Hedgehog (SHH) to the Patched tumor suppressor gene (PTCH) receptor. This initiates a series of events that results in the activation and nuclear translocation of the Gli family of transcription factors which regulate genes controlling proliferation, differentiation, survival and epithelial-mesenchymal transition. The HH signaling pathway is necessary for self-renewal and maintenance of stem cells^8^ and has been shown to promote proliferation of both mammary and BCSC.^9^ Increased levels of HH family members SHH, Gli-1 and Gli-2 have been reported in BCSC isolated from human tumors, compared to the bulk of tumor cells.^9,10^ We have previously reported that HH activation is increased in recurrent tumors after treatment with chemotherapy in a rat model of HER2-positive breast cancer.^11^ However, it is unknown if this is a result of preferential survival of resistant HH positive cells, or induction of HH activation upon chemotherapy induced damage.

Tissue damage due to chemotherapy can trigger an inflammatory reaction similar to a wound healing response resulting in increases in inflammatory signals. This can in turn reactivate developmental pathways that are necessary for wound closure and tissue regeneration, leading to a tumor promoting environment. In normal adult tissue, HH signaling is important for expansion of stem cell populations during tissue injury. Hedgehog signaling is induced by inflammatory factors at the site of injury and promotes plasticity of epidermal cells during re-epithelialization, leading to increases in stem cell populations.^12^ In addition, inhibition of hedgehog signaling has been shown to impair wound healing reactions by decreasing proliferation of progenitor populations.^12^ Likewise, we have previously reported that expansion of BCSC after taxane-based chemotherapy is dependent upon activation of the hedgehog signaling pathway.^13^ HH signaling was activated *in vitro* by chemotherapy treatment in breast cancer cell lines and inhibition of hedgehog signaling led to a decrease in expansion of stem-like populations and a decrease in clonogenic survival after treatment with docetaxel.

In this paper, we examine the kinetics of hedgehog signaling in a TNBC patient derived xenograft model of residual disease after treatment with docetaxel. We show that HH pathway activation occurs transiently after chemotherapy treatment, is correlated with release of inflammatory cytokines and precedes expansion of BCSC.

## METHODS

### Animal Model and Chemotherapy Treatment

All studies were conducted under an animal use and drug delivery protocol approved by the University of Delaware Institutional Animal Care and Use Committee (IACUC). Eight-week-old female Nonobese diabetic/severe combined immunodeficiency (NOD/SCID) Patient Derived Xenografts (PDX) tumor bearing mice with a P1-P3 fragment of a human patient derived breast cancer xenograft TM00089 implanted subcutaneously (The Jackson Laboratory) were obtained for use in the chemotherapeutic studies. Mice were housed in a barrier facility at the University of Delaware. Once tumors reached 4 mm in size, mice were randomly divided into 5 groups of 3 mice each. One group served as day 0 and was euthanized immediately. Three groups received weekly i.p. 0.5 ml injections of 15 mg/kg of docetaxel dissolved in 10% ethanol, 5% glucose in water to block tumor growth. Groups of mice were euthanized on post-docetaxel treatment day 2, 8, or 15. One group of mice were treated with weekly i.p. 0.5 ml injections of 15 mg/kg of docetaxel for 3 weeks. At post-docetaxel treatment day 21, treatment was stopped to monitor re-growth of tumor. Mice were monitored and tumor development was documented twice weekly by Vernier calliper measurements. Tumor volume was calculated as (length×width×width)/2. All mice were euthanized by CO_2_ asphyxiation followed by cervical dislocation and tumors were excised from each mouse. Tumors were fixed in formalin and then embedded in paraffin by the Histochemistry & Tissue Processing Core Lab of Nemours/Alfred I. duPont Hospital for Children. Longitudinal 5 μm-thick sections were obtained from each sample block and used for immunohistochemical staining.

### Immunohistochemistry

Slides were deparaffinized in Citrasolv (3×10 min) and rehydrated in ethanol at decreasing concentrations (100%, 90%, and 80% for 2×3 min each) ending in distilled water for 30 s. Slides were then heated in a microwave oven in 1x Citra for antigen retrieval. After cooling to room temperature, staining was done according to DAB Substrate Kit protocol (ab64238). Slides were washed with Phospate Buffered Saline (PBS) (2×2 min) and then incubated with peroxidase quenching solution for 5 minutes. Slides were washed with PBS (2×2 min) and then incubated with blocking solution for 10 minutes. Blocking solution was rinsed off and slides were incubated overnight at 4 °C with rabbit polyclonal antibodies against IL-6 or IL-8 (ab154367 1:100, ab106350 1:100), and rabbit monoclonal antibodies against Gli-1, Sonic Hedgehog, ALDH1A3, (ab53281 1:100, ab134906 1:100, ab52492 1:100). Slides were washed in PBS (2×2 min) and then incubated with biotinylated secondary antibody for 10 min. Slides were washed in PBS (2×2 min) and then incubated with streptavidin-peroxidase conjugate or 10 min. Slides were washed in PBS (2×2 min) and then incubated with 3,3’-Diaminobenzidine (DAB) chromogen for 5 min. Slides were washed in running deionized water for 2 minutes and then counterstained with hematoxylin for 2 minutes. Slides were washed with running deionized water for 2 minutes and then incubated in PBS for 30 s. Slides were dehydrated using an increasing ethanol concentration (70%, 80%, 95%, 100%) and then mounted. Slides were analyzed using Nikon Eclipse TS100 microscope. The number of positive tumor cells per 20X field were counted as a percentage of total cells. Five fields were counted per sample. Positive tumor cell counts were independently verified by multiple investigators blinded to the treatment order.

### Statistical Analysis

Statistical analysis was performed using Graph Pad Prism 6 software (Graph Pad, La Jolla, CA, USA). The mean values of data were evaluated using Analysis of variance (ANO-VA) followed by an unpaired test. For all tests, p values less than 0.05 were considered to be significant.

## RESULTS

### Hedgehog Activation Occurs Transiently after Treatment with Docetaxel and Proceeds Tumor Regrowth

**Figure 1:**
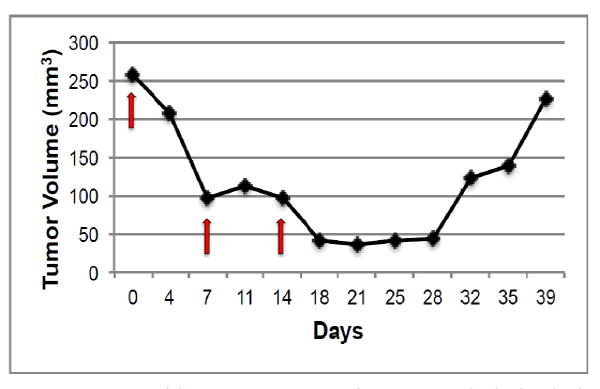

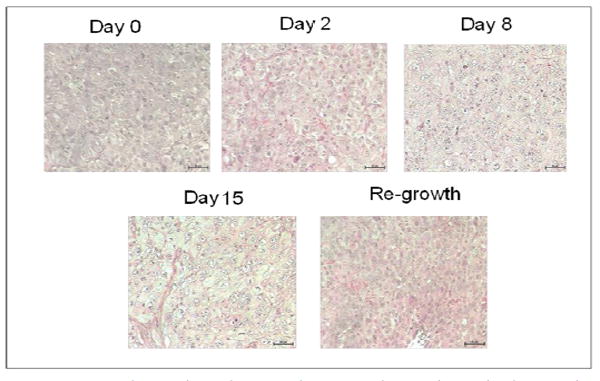
**A:** Representative graph of changes in tumor volume after treatment with docetaxel. Red arrows indicate administration of docetaxel. **B:** H&E stains of tumor tissues obtained prior to and at indicated time-points after docetaxel treatments. Images were acquired using a 20X objective.

**Figure 2:**
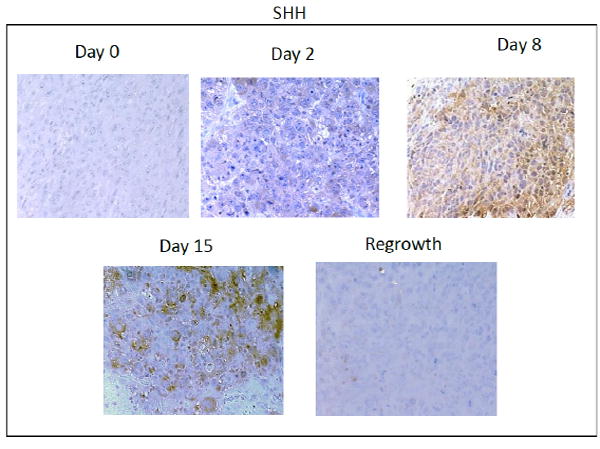

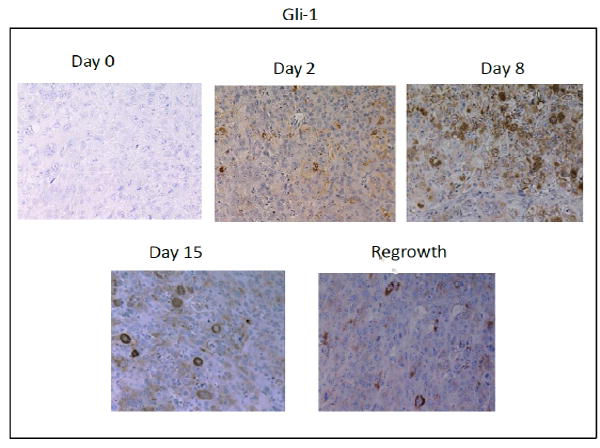

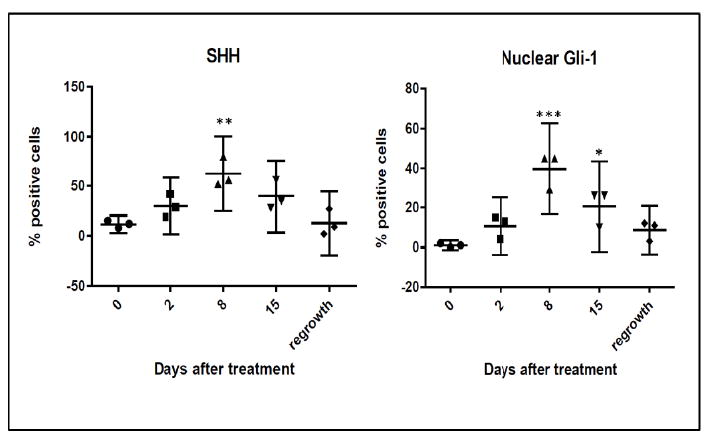
**A:** Immunohistochemical analysis of SHH expression in xenograft tissues prior to and at the indicated timepoints after docetaxel treatment. Images were acquired using a 20X objective. **B:** Immunohistochemical analysis of Gli-1 expression in xenograft tissues prior to and at the indicated timepoints after docetaxel treatment. Nuclear and cytoplasmic expression of Gli-1 is observed after treatment. Images were acquired using a 20X objective. **C:** Quantification of percent of cells positive for moderate to strong staining of SHH and Nuclear Gli-1 in five 20X fields. n=3 mice per time point. Error bars represent 95% confidence interval. Asterisk represent a significant statistical difference from untreated, *p<.01, **p<.005, ***p<.001.

In order to determine if HH activation occurs after *in vivo* treatment with chemotherapy, we chose to examine the kinetics of HH activation in a patient derived tumor xenograft mouse model that was essentially negative by immunostaining for expression of HH family members prior to treatment. Upon weekly injections of 15 mg/kg of docetaxel, we observed a decrease in tumor volume beginning around day 4 post-docetaxel treatment and reaching a nadir between 14-18 days after treatment at which time an increase in tumor volume was observed (Figure 1). Tumors reached their pre-treatment size approximately 35-39 days after the start of treatment. Prior to chemotherapy treatment, there was minimal expression of SHH and nuclear Gli. Increases in both SHH and nuclear Gli-1 expression were observed within 48 hours after treatment with chemotherapy, peaking around day 8 when approximately 63% (range 52-80%, SD 12.0) of cells were positive for expression of SHH. This correlated with nuclear Gli-1 expression in approximately 40% (range 29-45%, SD 6.1) of cells, indicating full activation of HH signaling. Levels of SHH and nuclear Gli-1 began to decrease after day 8, remaining only slightly above pre-treatment levels upon tumor regrowth at day 39 (Figure 2). These findings indicate that HH signaling, including ligand release and nuclear Gli-1 translocation, are transiently induced by chemotherapy, and may promote tumor regrowth.

### Hedgehog Activation after Docetaxel Treatment Correlates with Inflammatory Responses

**Figure 3:**
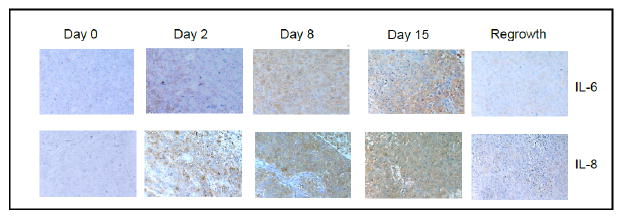

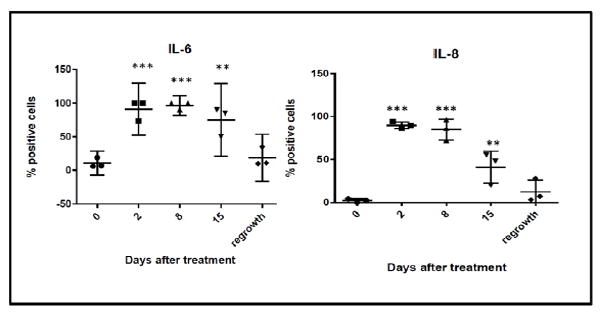
**A:** Immunohistochemical analysis of IL-6 and IL-8 expression in xenograft tissues prior to and at the indicated timepoints after docetaxel treatment. Images were acquired using a 20X objective. **B:** Quantification of percent of cells positive for moderate to strong staining of Il-6 and IL-8 in five 20X fields. n=3 mice per time point. Error bars represent 95% confidence interval. Asterisk represent a significant statistical difference from untreated, **p<.005, ***p<.001.

During the normal wound healing process, HH activation is induced by the inflammatory response within the site of tissue injury. Inflammatory cytokines such as interleukin-6 (IL 6) and interleukin-8 (IL-8) are released during tissue injury and help to initiate re-epithelialization.^14^ We sought to determine if release of these cytokines correlated with HH expression in our model of residual disease. Similar to the expression of SHH and nuclear Gli-1, we observed transient increases in IL-6 and IL-8 within 48 hours after chemotherapy treatment, peaking around day 8 post-docetaxel treatment (Figure 3). Levels of IL-6 and IL-8 continued to decrease upon tumor regrowth with no significant difference between observed pre-treatment levels and those at day 39.

### Sustained Increases in ALDH1A3 Positive Populations Occur after Chemotherapy

**Figure 4:**
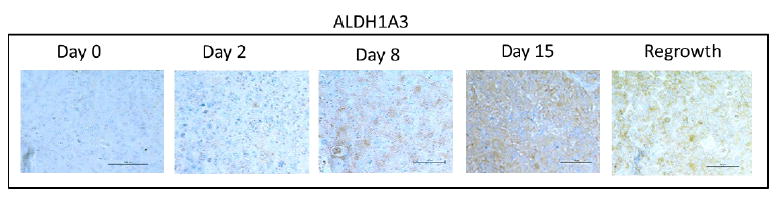

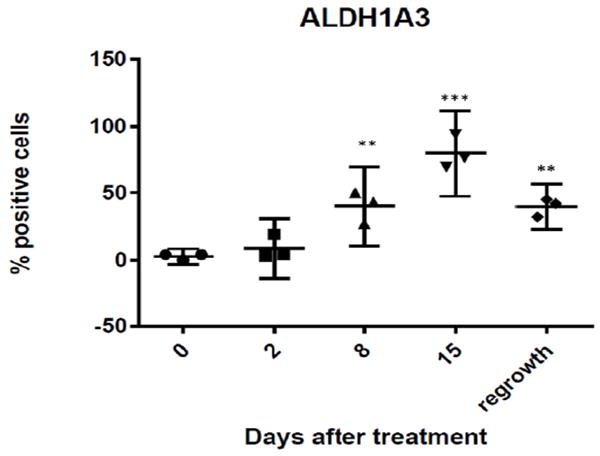
**A:** Immunohistochemical analysis of ALDH1A3 expression in xenograft tissues prior to and at the indicated timepoints after docetaxel treatment. Images were acquired using a 20X objective. **B:** Quantification of percent of cells positive for moderate to strong staining of ALDH1A3 in five 20X fields. n=3 mice per time point. Error bars represent 95% confidence interval. Asterisk represent a significant **p<.005, ***p<.001.

We have previously shown that HH activation is induced after chemotherapy and promotes increases in stem-like populations in breast cancer cell lines.^13^ Likewise, both IL-6 and IL-8 have been linked to expansion of stem-like populations after chemotherapy treatment.^15^ Consistent with other studies, we showed an increase of cells expressing the stem-cell marker AL-DH1A3 following docetaxel treatment (Figure 4). This increase occurred at day 15, and was preceded by increased expression of inflammatory cytokines and activation of HH signaling, suggesting that these signals promote an expansion of ALDH1A3 cells. Although, there was a slight decrease in ALDH1A3 positive cells at regrowth, in contrast to the transient expression observed for the inflammatory markers, expression of ALDH1A3 remained significantly increased at regrowth compared to pre-treatment levels (*p*<0.005). These data indicate a long-term expansion of the ALDH1A3 positive cell population.

## DISCUSSION

Our previous studies have shown that HH signaling is increased in recurrent tumors and may promote expansion of BCSC after treatment. The results of our current study suggest that rather than an expansion of resistant HH positive cells, HH signaling is induced by chemotherapy and promotes expansion of ALDH1A3 positive BCSC. These findings are consistent with our previous studies, which showed that HH signaling was induced in the majority of tumor cells, but did not provide a protective effect. Rather, HH signaling may have an effect on a small population of resistant cells, which then may repopulate the tumor.

Our data further demonstrates that inflammatory processes are activated after treatment with chemotherapy. During tissue injury, inflammatory cytokines such as IL-6 and IL-8 are released shortly following wounding. This inflammatory response may initiate events that result in the activation of HH signaling, which then promotes expansion of progenitor populations, leading to re-epithelialization of the wound.^16-18^ Similar to our findings in the current study, levels of IL-6 and IL-8 at the injury site are transient and decrease following re-epithelialization.^19^ These findings suggest that a wound healing reaction occurs as a result of tissue damage following chemotherapy, which may trigger activation of pathways such as HH by the dying tumor cells. As in tissue injury, these signals may promote expansion of stem-like cells, leading to regrowth of the tumor. In our model, this inflammatory reaction is transient. However, the increases in the population of stem-like cells remain after tumor regrowth and may promote further resistance or metastasis after treatment.

Both IL-6 and IL-8 have been found to promote an aggressive tumor microenvironment through activation of the STAT-3-NfKB signaling. Like HH activation, activation of STAT-3 has been shown to increase proliferation of BCSCs.^20^ IL-6-STAT3 signaling is reported to indirectly upregulate canonical hedgehog signaling in other tumor models.^21^ Whether the increase of SHH observed in our model after chemotherapy is directly or indirectly regulated by IL-6-STAT3 signaling is unknown. Likewise, it is possible that there may be significant cross-talk between the two pathways that promote increases in BCSCs.

It is unclear if the expansion of ALD1A3 cells following chemotherapy results from a proliferation of existing BCSCs, or the acquisition of a stem-like phenotype by differentiated tumor cells. While HH signaling has been shown to promote proliferation of both normal and cancer stem cells, it has also been shown to alter plasticity of committed cells. For example, during tissue injury, HH signaling has been shown to transform committed hair follicular cells into epidermal stem cells, whose progeny exhibit the features of self-renewing epidermal stem cells.^17,22^ In addition, changes in cellular plasticity have been reported after treatment of breast cancer cells with radiation. ALDH negative cells were shown to acquire an ALDH positive phenotype following treatment with high doses of ionizing radiation.^23^ It is possible that changes to the microenvironment of the stem cell niche during chemotherapy may promote de-differentiation of tumor cells. Additional confirmation of this BCSC expansion using other stemness markers is needed. Likewise, further investigation using lineage tracing experiments should be performed to determine if changes in cellular plasticity occur after chemotherapy, and if these can be prevented with the addition of HH inhibitors or anti-inflammatory agents.

## ABBREVIATIONS

TNBC: Triple Negative Breast Cancer

HER2: Human epidermal growth factor receptor 2

IL-6: Interleukin 6

IL-8: Interleukin 8

BCSC: Breast Cancer Stem Cells

DNA: Deoxyribonucleic acid

ALDH: Aldehyde dehydrogenase

HH: Hedgehog

PTCH: Patched tumor suppressor gene

SHH: Sonic Hedgehog

IACUC: Institutional Animal Care and Use Committee

NOD/SCID: Nonobese diabetic/severe combined immunodeficiency

PDX: Patient Derived Xenografts

DAB: 3,3’-Diaminobenzidine

PBS: Phospate Buffered Saline

ANOVA: Analysis of variance

## References

[1] Hudis CA, Gianni L (2011). Triple-negative breast cancer: an unmet medical need. Oncologist.

[2] Liedtke C, Mazouni C, Hess KR (2008). Response to neoadjuvant therapy and long-term survival in patients with triple-negative breast cancer. J Clin Oncol.

[3] Yu KD, Zhu R, Zhan M (2013). Identification of prognosis-relevant subgroups in patients with chemoresistant triple-negative breast cancer. Clin Cancer Res.

[4] Marcato P, Dean CA, Pan D (2011). Aldehyde dehydrogenase activity of breast cancer stem cells is primarily due to isoform ALDH1A3 and its expression is predictive of metastasis. Stem Cells.

[5] Honeth G, Lombardi S, Ginestier C (2014). Aldehyde dehydrogenase and estrogen receptor define a hierarchy of cellular differentiation in the normal human mammary epithelium. Breast Cancer Res.

[6] Yasmeen R, Reichert B, Deiuliis J (2013). Autocrine function of aldehyde dehydrogenase 1 as a determinant of diet- and sex-specific differences in visceral adiposity. Diabetes.

[7] Marcato P, Dean CA, Liu RZ (2015). Aldehyde dehydrogenase 1A3 influences breast cancer progression via differential retinoic acid signaling. Mol Oncol.

[8] Ingham PW, McMahon AP (2001). Hedgehog signaling in animal development: paradigms and principles. Genes Dev.

[9] Liu S, Dontu G, Mantle ID (2006). Hedgehog signaling and Bmi-1 regulate self-renewal of normal and malignant human mammary stem cells. Cancer Res.

[10] Shipitsin M, Campbell LL, Argani P (2007). Molecular definition of breast tumor heterogeneity. Cancer cell.

[11] Smith D, Kong F, Yang D, Larson R, Sims-Mourtada J, Woodward WA (2014). Patched targeting peptides for imaging and treatment of hedgehog positive breast tumors. Biomed Res Int.

[12] Le H, Kleinerman R, Lerman OZ (2008). Hedgehog signaling is essential for normal wound healing. Wound Repair Regen.

[13] Sims-Mourtada J, Opdenaker LM, Davis J, Arnold KM, Flynn D (2014). Taxane-induced hedgehog signaling is linked to expansion of breast cancer stem-like populations after chemotherapy. Mol Carcinog.

[14] Werner S, Grose R (2003). Regulation of wound healing by growth factors and cytokines. Physiol Rev.

[15] Ginestier C, Liu S, Diebel ME (2010). CXCR1 blockade selectively targets human breast cancer stem cells in vitro and in xenografts. J Clin Invest.

[16] Tierney MT, Aydogdu T, Sala D (2014). STAT3 signaling controls satellite cell expansion and skeletal muscle repair. Nat Med.

[17] Levy V, Lindon C, Zheng Y, Harfe BD, Morgan BA (2007). Epidermal stem cells arise from the hair follicle after wounding. FASEB J.

[18] Arnold KM, Opdenaker LM, Flynn D, Sims-Mourtada J (2015). Wound healing and cancer stem cells: inflammation as a driver of treatment resistance in breast cancer. Cancer Growth Metastasis.

[19] Kondo T, Ohshima T (1996). The dynamics of inflammatory cytokines in the healing process of mouse skin wound: a preliminary study for possible wound age determination. Int J Legal Med.

[20] Korkaya H, Liu S, Wicha MS (2011). Regulation of cancer stem cells by cytokine networks: attacking cancer’s inflammatory roots. Clin Cancer Res.

[21] Yang Q, Shen SS, Zhou S, Ni J, Chen D, Wang G, Li Y (2012). STAT3 activation and aberrant ligand-dependent sonic hedgehog signaling in human pulmonary adenocarcinoma. Exp Mol Pathol.

[22] Brownell I, Guevara E, Bai CB, Loomis CA, Joyner AL (2011). Nerve-derived sonic hedgehog defines a niche for hair follicle stem cells capable of becoming epidermal stem cells. Cell Stem Cell.

[23] Lagadec C, Vlashi E, Della Donna L, Dekmezian C, Pajonk F (2012). Radiation-induced reprogramming of breast cancer cells. Stem Cells.

